# The potential effect of natural antioxidants on endothelial dysfunction associated with arterial hypertension

**DOI:** 10.3389/fcvm.2024.1345218

**Published:** 2024-02-02

**Authors:** Rosamaria Caminiti, Cristina Carresi, Rocco Mollace, Roberta Macrì, Federica Scarano, Francesca Oppedisano, Jessica Maiuolo, Maria Serra, Stefano Ruga, Saverio Nucera, Annamaria Tavernese, Micaela Gliozzi, Vincenzo Musolino, Ernesto Palma, Carolina Muscoli, Speranza Rubattu, Maurizio Volterrani, Massimo Federici, Massimo Volpe, Vincenzo Mollace

**Affiliations:** ^1^ Department of Health Sciences, Institute of Research for Food Safety and Health (IRC-FSH), University “Magna Graecia” of Catanzaro, Catanzaro, Italy; ^2^ Department of Health Sciences, Veterinary Pharmacology Laboratory, Institute of Research for Food Safety and Health (IRC-FSH), University “Magna Graecia” of Catanzaro, Catanzaro, Italy; ^3^ Department of Systems Medicine, University “Tor Vergata” of Rome, Rome, Italy; ^4^ Laboratory of Pharmaceutical Biology, Department of Health Sciences, Institute of Research for Food Safety and Health (IRC-FSH), University “Magna Graecia” of Catanzaro, Catanzaro, Italy; ^5^ IRCCS San Raffaele Roma, Rome, Italy; ^6^ IRCCS Neuromed, Pozzilli, Italy; ^7^ Department of Clinical and Molecular Medicine, School of Medicine and Psychology, Sapienza University, Rome, Italy; ^8^ Renato Dulbecco Institute, Catanzaro, Italy

**Keywords:** natural antioxidants, arterial hypertension, cardiovascular disease, endothelial dysfunction, ROS, NO

## Abstract

Arterial hypertension represents a leading cause of cardiovascular morbidity and mortality worldwide, and the identification of effective solutions for treating the early stages of elevated blood pressure (BP) is still a relevant issue for cardiovascular risk prevention. The pathophysiological basis for the occurrence of elevated BP and the onset of arterial hypertension have been widely studied in recent years. In addition, consistent progress in the development of novel, powerful, antihypertensive drugs and their appropriate applications in controlling BP have increased our potential for successfully managing disease states characterized by abnormal blood pressure. However, the mechanisms responsible for the disruption of endogenous mechanisms contributing to the maintenance of BP within a normal range are yet to be fully clarified. Recently, evidence has shown that several natural antioxidants containing active ingredients originating from natural plant extracts, used alone or in combination, may represent a valid solution for counteracting the development of arterial hypertension. In particular, there is evidence to show that natural antioxidants may enhance the viability of endothelial cells undergoing oxidative damage, an effect that could play a crucial role in the pathophysiological events accompanying the early stages of arterial hypertension. The present review aims to reassess the role of oxidative stress on endothelial dysfunction in the onset and progression of arterial hypertension and that of natural antioxidants in covering several unmet needs in the treatment of such diseases.

## 1 Introduction

Maintaining normal BP has been shown to significantly decrease the risk of cardiovascular events, including myocardial infarction, stroke, and chronic kidney disease (CKD) (1). The central mechanisms regulating blood pressure, liquid and electrolyte balance, endocrine factors, and local mechanisms that modulate vascular tone have all been implicated in the pathophysiological mechanisms underlying the development of arterial hypertension (2). However, the mechanisms that play a key role in the development of arterial hypertension during the early stages of the disease and their optimal treatment still remain to be clarified. Endothelial cells were shown to be able to generate vasoactive products, radicals, and molecules that enable the maintenance of proper vascular tone, the physiological course of hemostasis, and angiogenesis, thereby contributing to the regulation of endocrine and metabolic functions (3). All of the above are involved in preventing atherosclerosis and thrombosis (4); in particular, evidence shows that nitric oxide (NO) and a healthy endothelium are able to maintain normal BP (5). Hypertension has been shown to be closely associated with an increase in free radicals, which counteracts normal endothelial performance (6). Different therapies are currently used to counteract the onset and progression of hypertension resulting from endothelial dysfunction (7, 8). Nevertheless, approximately 40% of hypertensive patients showed resistance to traditional pharmacological therapies (9–11). This implicates the presence of other molecular pathways, including oxidative stress and inflammation, in hypertension development and progression, suggesting the need to investigate the potential role of natural antioxidants as adjuvants to antihypertensive drugs. For this reason, this review aims to evaluate the potential effect exerted by free radical species generated by the vascular wall on hypertension and, in particular, whether supplementation with natural antioxidants can maintain endothelial health and positively affect blood pressure. In particular, we will describe the key role of nitrosative and oxidative stress in promoting the transition from normal BP to hypertension. Finally, we will conclude with a description of the underlying mechanisms of selected nutraceuticals with antioxidant and antihypertensive activities that were recently assessed in clinical trials.

## 2 Role of a healthy endothelium in the regulation of blood pressure

### 2.1 The endothelial cell layer

While until the early 1980s it was believed that the endothelium had a solely passive role in organizing the vascular envelope and managing the selective passage of water and electrolytes alone, it is now known that this tissue performs numerous other vital functions. These involve vascular quiescence, vascular tone and structure regulation, angiogenesis, the control of hemostasis and inflammation, endocrine–metabolic functions, and protection from tissue and ischemic injuries (3, 12). The endothelium is able to release a large variety of molecules, including vasoactive substances, growth factors, adhesion molecules, inflammation mediators, and proteins involved in the hemostatic process, into both the blood and the interstitial space. Taken together, these mechanisms are responsible for endothelial homeostasis and its autocrine activity since they can act at a distance, conferring endocrine activity on the endothelium itself and paracrine activity on nearby cells (13). The balance obtained results in an adequate immune response. Reduced or inadequate vasodilation, on the other hand, will eventually lead to common heart conditions, including hypertension, peripheral vascular disease, coronary artery disease, diabetes, chronic renal failure, or different viral infections (14).

### 2.2 Role of NO in endothelium-dependent vasodilation

NO is a small molecule with a few seconds of half-life, is involved in cardiac contractility regulation, and is at the forefront of the pathway that regulates blood vessel dilation. It leads to an increase in blood supply with the consequent BP reduction (15). Indeed, NO regulates blood flow supply to the cardiovascular and renal systems. In the first case, NO prevents platelet aggregation, smooth muscle cell proliferation, and leukocyte adhesion. On the renal level, it promotes diuresis and natriuresis (16). In healthy endothelial cells, NO is the reaction product between L-arginine and molecular O_2_, catalyzed by NO synthase (NOS), which consists of three isoforms: endothelial (eNOS), inducible (iNOS), and neuronal (nNOS). The activity of eNOS is regulated by several partners, such as heat shock protein 90 (HSP90), calmodulin (CaM), eNOS-interacting protein (NOSIP), and caveolin-1 (Cav-1). Studies on Cav-1 knockout mice demonstrated increased levels of eNOS and subsequent vasorelaxation. Indeed, the reduction in caveolin levels attenuates hypertension caused by angiotensin II (Ang II) uptake via the Ang II type I receptor (AT1) at the renal proximal tubule (17). Kleschyov (18) suggests that it is not the free NO that is released by NOS but rather the whole heme–NO complex. Indeed, it has been shown that NO, produced by NOS, through binding with cytosolic heme, forms a complex that triggers the assembly and activation of guanylate cyclase (sGC) (19). Furchgott and Zawadzki (20) demonstrated, for the first time, how the removal of the endothelium from vascular preparations *in vitro* can prevent Ach-induced vasodilatation, thus proposing CaM as the *primum movens* of eNOS activation; in fact, CaM could be regulated by intracellular calcium flux modifications and mobilization. Furthermore, it appears that heat shock protein 90 could play a role in the regulation of CaM sensitivity toward the eNOS enzyme (21).

**Figure 1:**
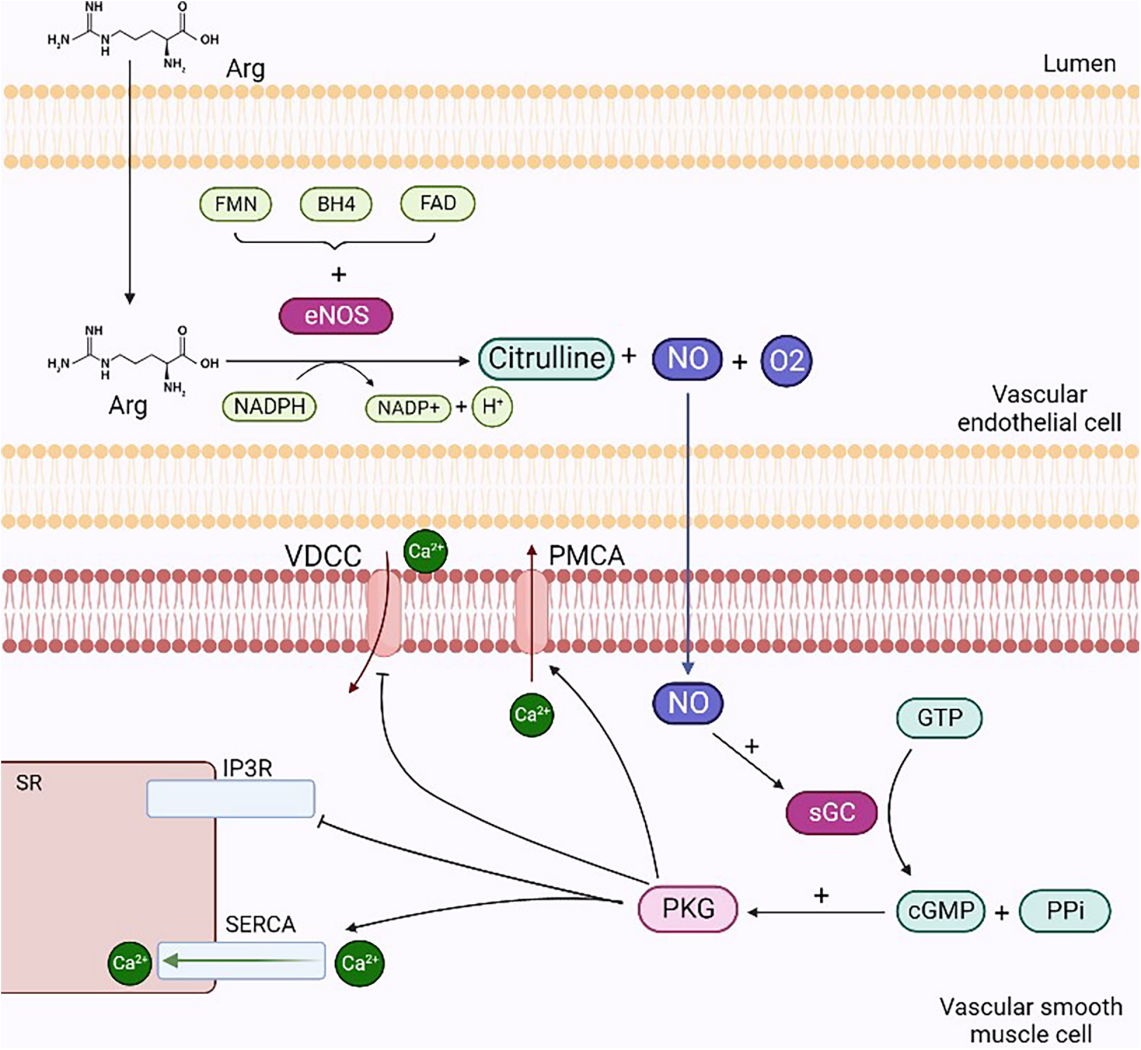
Mechanisms of NO-mediated vasodilation. NO produced through the degradation reaction of L-arginine into citrulline diffuses from endothelial cells to smooth muscle cells and activates sGC by forming cGMP and PPi. cGMP activates PKG, which stimulates the opening of SERCA and PMCA, enabling the reduction in intracellular calcium concentrations: in the former case, ions enter the interior of the SR, while in the latter case, they escape from the cell. Finally, PKG inhibits IP3r and VDCC, preventing calcium efflux from the SR and ion entry into the cell, respectively. Arg, arginine; VDCC, voltage-dependent calcium channels; PMCA, plasma membrane ATPases; sGC, guanylate cyclase; PPi, inorganic pyrophosphate; PKG, protein kinase G; SERCA, sarcoplasmic calcium ATPases; IP3R, inositol trisphosphate receptors. Created with BioRender.com.

The posttranslational regulation of eNOS is dependent on protein kinase C (PKC), and during an inflammatory state, it is mediated by Rho-kinase. PKC phosphorylation occurs in all endothelial cells with a negative regulatory significance. In particular, the inhibition of eNOS results from the PKC phosphorylation of Thr495, amplified by the simultaneous dephosphorylation of Ser-1177 (22). eNOS is constitutively expressed in endothelial cells and is mainly located in the Golgi apparatus, peri-nucleus, and caveolae (23). Schlaich et al. (24) showed that the seizure of Arg in intracellular compartments inaccessible to eNOS and the compartmentation of the enzyme within the caveolae can significantly limit NO production. In this study, hypertensive and normotensive subjects who were genetically predisposed to positive hypertension were compared with subjects with a family history of negative hypertension. Both groups were radiomarked with Arg via intra-arterial infusion, and blood venous samples were analyzed. The results obtained showed that the subjects with a family history of negative hypertension were unable to maintain significant intracellular concentrations of Arg. The use of mice with alteration of the eNOS gene stresses the role of this enzyme on vasodilatation. This alteration caused an increase of 20 mmHg in mean pressure if compared with that of wild-type mice in the waking state (15). iNOS results are upregulated during the inflammatory state, which can be induced by LPS stimulation or endogenous cytokine production. iNOS could lead to endothelial dysfunction and, finally, to atherothrombosis. Furthermore, it causes a constant, albeit delayed, release of a significant amount of NO, which triggers apoptosis as a defense mechanism, contributing to the pathogenesis of chronic diseases (25). NO produced via iNOS catalysis results in elevated levels of peroxynitrite, a radical that is harmful to health as it leads to the apoptosis of endothelial cells. Finally, nNOS is involved in astrocyte and neuron neurotransmission (16). It is expressed in the peripheral and central nervous systems and is upregulated in hypertension (26). Once produced, NO spreads to the underlying vascular smooth muscle cells (VSMCs) and activates sGC. These events, through specific cascade activation, lead to calcium efflux prevention from the sarcoplasmic reticulum (SR) and calcium entry into the cell. The final outcome is vasodilatation, since the reduction in calcium concentration prevents the activation of myosin light chain kinase through the calcium–calmodulin complex while upregulating, however, the acitivity of myosin light chain phosphatase (Figure 1) (15).

### 2.3 Other players involved in endothelium-dependent vasodilatation and vasoconstriction

Endothelium-derived contraction factors include Ang II, thrombin, thromboxane A2 (TxA2), O_2_^−^, and endothelin 1 (ET-1) (14). The latter is generated by endothelial cells as a result of different stimuli, such as the presence of reactive oxidative species (ROS), inflammation, and adhesion molecules. It induces vasoconstriction by activating ETA and ETB2 receptors on smooth muscle cells, increases calcium concentration in intracellular compartments, and subsequently triggers myosin light chain activation. However, ET-1 binds endothelial membrane ETB1 receptors, activating a signaling cascade leading to the production of NO and PGI2 and, finally, vascular relaxation (27). This different behavior regarding the activity of ET-1 is related to its concentration: reduced levels of ET-1 result in vasodilatation, while increased levels lead to peripheral vascular resistance and high BP. Indeed, ET-1 is actively involved in the arterial remodeling process and causes the thickening of small arteries through hypertrophy. The combination of vascular wall thickness and tone determines the increase in peripheral vascular resistance, which is a distinctive trait of hypertension onset (28). In addition, ET-1 induces inflammation by recruiting and activating immune cells in the bloodstream (29) and by promoting NOX expression in vascular cells. ROS production causes the synthesis and release of inflammatory molecules, including cytokines and adhesion molecules (30). Another important molecule for maintaining BP is the angiotensin-converting enzyme (ACE). Angiotensin, once converted into Ang II, interacts with the AT1 receptor, leading to the synthesis of aldosterone, vasopressin, prolactin, and ROS. In addition, Ang II production induces the release of different molecules (ET-1, adhesion molecules, and growth factors), resulting in sodium and water retention and initiating endothelial impairment. Consequently, this leads to increased vasoconstriction, inflammation, and remodeling of resistant arteries. Moreover, Ang II leads to myocyte hypertrophy, fibrosis, and ultimately hypertension. In contrast, when the same molecule binds Ang II type II (AT2) receptors, the effects produced conflict with the ones described above; they result, in fact, in homeostatic effects and in vasodilation states. However, because Ang II has greater affinity for the first receptor, the corresponding pathway is prevalent, with consequent detrimental effects on BP (31). The spread of SARS-CoV-2 has drawn attention to a second isoform of this enzyme, known as ACE2. ACE2 converts Ang II into Angiotensin 1-7, which interacts with the MAS receptor on endothelial cells, achieving an increase in NO availability, ROS reduction, and positive effects on BP (32).

### 2.4 Regulation of the endothelium integrity: the role of endothelial glycocalyx on NO bioavailability

Macromolecules can cross the endothelial barrier through cell–cell junctions, diffusion, vesicular transport, endothelial gaps, or through the cells themselves. Moreover, different endothelial cell microstructures are important for the integrity of endothelial cells. Several of these, including proteoglycan core proteins, sialo-glycoproteins, surface glycoproteins, and glycosaminoglycans (GAGs), constitute endothelial glycocalyx and are essential for tissue integrity (14). The relationship between endothelial health and NO availability is significantly influenced by the endothelial glycocalyx, which represents a mediator for NO production itself. The endothelial glycocalyx decay leads to a decrease in NO production by endothelial cells in response to fluid shear stress (33). The glycocalyx is involved in endothelial phenotype preservation and regulates flow-dependent NO production, trans-endothelial permeability, leukocyte adhesion, and passage through the endothelium. Heparan sulfate is the major endothelial GAG and is covalently linked to proteoglycan core proteins: transmembrane syndecans (such as SDC-1) and membrane-bound glypican (GPC-1). Heparan sulfate, together with GPC-1 and SDC-1, is produced and released by endothelial cells following flow alterations. In particular, GPC-1 is located within the caveolae, where it interacts with eNOS and other flux-regulated signaling molecules (34). eNOS expression is regulated by the glycosaminoglycan heparan sulfate and the proteoglycan core protein glypican-1. Ebong et al. (34) demonstrated that the knockdown of GPC1 in bovine aortic endothelial cells, and a minor one in SDC-1, leads to the inhibition of shear-induced eNOS phosphorylation. Specifically, since phosphorylated eNOS levels remain elevated after only SDC-1 knockdown, the inhibition is due to GPC-1 suppression. These data are confirmed by the study conducted by Bartosch and colleagues (33). They demonstrated, for the first time, the NO production by specific glycocalyx structures. In particular, they used antibody-functionalized atomic force microscopy probes on both GPC-1 and SDC-1 for 10 min, showing that NO levels were increased in the former case. Therefore, it is possible to consider GPC-1 as the main regulator of the flux-induced eNOS phosphorylation, and there is a need to identify treatments for the maintenance of GPC-1 in the glycocalyx, a structure that is often underestimated but represents an important contribution to endothelial health. Under conditions of intact glycocalyx, regular flow, and elevated physiological shear stress, endothelial permeability appears to be reduced. On the other hand, the latter increases when the flow is altered, with decreased physiological shear stress and a low component of heparan sulfate and sialic acids (34). Salt overload can lead to glycocalyx degradation and consequent endothelial dysfunction; the glycocalyx buffering capacity decreases, the amount of sodium able to reach the endothelial cells increases, as well as the adhesion forces between endothelial surfaces and monocytes, resulting in vascular inflammation (35). Other stimuli may cause acute and chronic alterations in endothelium permeability, such as the vascular endothelial growth factor (VEGF), thrombin, and histamine release, as well as acute inflammation regulators such as tumor necrosis factor-α (TNF-α) and interleukin 1b (IL-1b). Excessive endothelial permeability and increased Ang II levels can arise from high levels of inflammation and oxidative stress, leading to the reduced availability of NO and the disruption of proteins involved in adherens junctions, tight junctions, and gap junctions (36). RNS and ROS are able to break the glycocalyx GAGs, resulting in modifications of the saccharide residues. Hydrolytic cleavage leads to unstable constituents obtaining, thus, polysaccharide fragmentation (37). Furthermore, sepsis status leads to pro-oxidant events, where ROS production is increased by leukocytes and endothelial cells. Overall, these factors induce glycocalyx deterioration, increased permeability, and endothelial dysfunction (38). Oxidative stress actively participates in the alteration of the glycocalyx. ROS cause a decrease in tissue inhibitors of metalloproteinases (TIMPs), thus resulting in high levels of metalloproteases (MMPs). These enzymes cleave the syndecan core protein, resulting in the alteration of the vascular walls, inflammation, and thrombosis. Hyaluronidase degrades hyaluronic acid, the byproducts of which enable ROS production. A vicious circle is therefore created as the latter depolymerizes the hyaluronic acid itself. Finally, heparanase cleaves the heparan sulfate side chains; this decreases extracellular SOD, the overexpression of which attenuates that of heparanase. This suggests that the preventive antioxidant strategy is functional to obviate the degradation of the glycocalyx (39). In addition, several studies highlighted the interdependent activity of glycocalyx and epithelial Na^+^ channels (ENaC). In particular, recent experimental data showed that the physical connection (based on N-glycans attached to glycosylated asparagines of α-ENaC) between glycocalyx and ENaC plays a crucial role in shear stress sensing; thus, specific N-glycan removal is able to downregulate the shear stress response. Another experimental study demonstrated the blood pressure increase in animals transduced with an endothelial-specific viral transduction of α-ENaC, while an attenuation of blood pressure increase has been observed in animals transduced with α-ENaC versions lacking N-glycans (40).

### 2.5 Exosome's role in endothelial function

**Figure 2:**
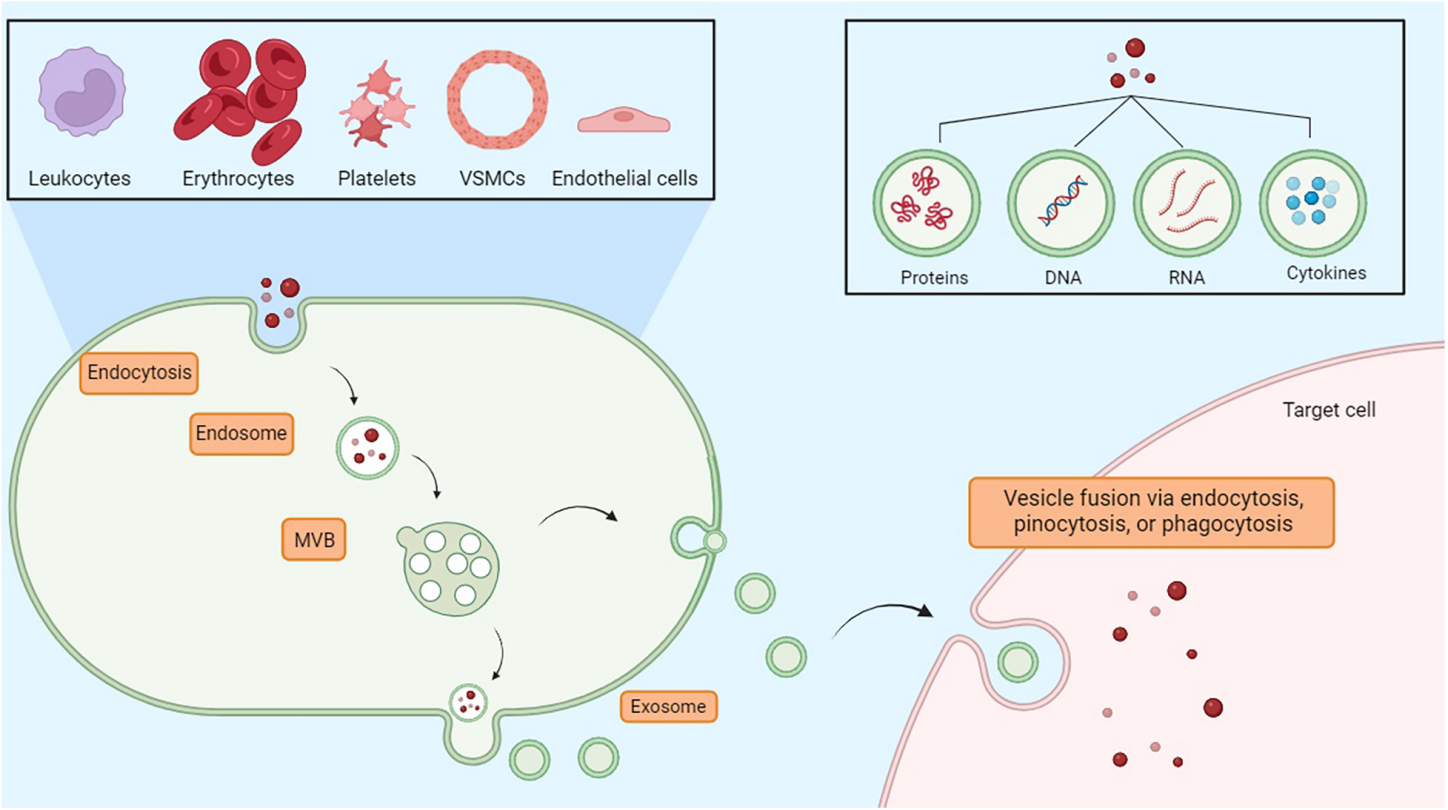
Exosomes are essential vesicles for communication among nearby or far-off cells. They are involved in inflammation control, vascular permeability, new blood vessel formation, and maintaining vascular balance. MVB, multivesicular body.

Exosomes are small vesicles that play an important role in the endothelial tissue, allowing intercellular communication and contributing to different physiological processes. Indeed, cells secrete exosomes, which transport bioactive molecules such as nucleic acids (DNA, RNA, mRNA, miRNA), as well as proteins, cytokines, and lipids (41). In endothelial tissue, exosomes act as messengers, transporting these bioactive molecules to neighboring or distant cells, immune cells, or smooth muscle cells, thereby influencing cellular functions. Exosomes contribute to the regulation of the inflammation state, vascular permeability, angiogenesis, and vascular homeostasis (42). Therefore, in addition to their role in intercellular communication, they may represent diagnostic markers and potential therapeutic targets for endothelium-related disorders such as cardiovascular disease, inflammation-driven conditions, and vascular dysfunction (43). Exosomes originate by budding on the internal side of the multivesicular body (MVB) membrane, a process mediated by the lipid ceramide or by the endosomal sorting complex required for transport (ESCRT). Vesicles and target cells interact in different ways. Exosomes are released into the extracellular space through the fusion of the cellular membrane and the MVB. Following this stage, the vesicles subsequently deliver their contents through a new process of fusion with the target cell via phagocytosis, endocytosis, or pinocytosis. Fusion depends on the lipid composition of the vesicle membrane, and phosphatidylserine appears to contribute to this process (44). Moreover, the vesicles could also directly activate the surface receptors of the target cells through bioactive proteins and ligands (45). Through the transfer regulation of bioactive molecules, exosomes significantly contribute to the modulation of endothelial inflammatory processes, thereby affecting vascular function and the pathogenesis of inflammatory conditions related to endothelial dysfunction (Figure 2) (46).

Extracellular vesicles are involved in endothelial health preservation, contributing to both advantageous and disadvantageous processes. In particular, they play a role in vascular protection by reducing endothelial cell apoptosis through the p38 inhibition. Also, they promote endothelial repair through SPRED-1 inhibition via mRNA-126. However, extracellular vesicles impair vascular relaxation, decrease NO production by lowering eNOS phosphorylation, boost NADPH activity, and increase oxidative stress (47); in addition, by transferring miRNA-222, exosomes decrease the endothelial expression of ICAM-1 (48). Furthermore, depending on where the vesicles originate, they contribute to inflammation by releasing pro-inflammatory cytokines, transmigration, and monocyte adhesion following the increased expression of adhesion molecules. In fact, exosomes derived from monocytes that transfer inflammatory miRNAs to endothelial cells increase ICAM-1, IL-6, and CCL2 expressions and promote endothelial apoptosis (49). Exosomes secreted by endothelial cells stimulated by shear stress or transduced by KLF-2 contribute to atheroprotection by carrying miRNA 143/145. Those deriving from leukocytes and platelets and containing miRNA-223 penetrate the vascular wall, inhibit the proliferation and migration of VSMCs, and reduce the plaque size. However, unstable plaques are characterized by the large-quantity release of extracellular vesicles from VSMCs, leukocytes, and erythrocytes from the necrotic nucleus. They express surface antigens that induce T-cell proliferation, carry the catalytically active TNF-α converting enzyme, and promote the inflammatory response (46, 50). Lin et al. studied the exosomes’ crucial function on the endothelium, demonstrating that miR-92a, a nucleotide derived from endothelial cells, was able to negatively influence their activity, affecting migration and proliferation. The correlation between this miRNA and the transcription factors KFL-2 and KFL-4 has been highlighted in a balloon injury model and in HCAEC treatment. In particular, it has been observed that KFL-2 is involved in antithrombotic, anti-inflammatory, and antimigratory activities. Chrysin, a type of flavone found in several plants such as *Cytisus villosus* Pourr., *Passiflora caerulea* L., *Passiflora incarnata* L., *Hyphaene thebaica* L., and *Matricaria chamomilla* L., as well as in honey and propolis, could contribute to upregulating KLF2, thus decreasing miR-92a secretion from exosomes and improving intercellular communication (51, 52). Further studies demonstrated that the exosomes are involved in the miRNA transport during a myocardial infarction; indeed, once released into circulation, miRNAs are carried to the bone marrow, inactivate CXCR4 present on monocytes and lymphocytes, and boost the circulating bone marrow stem cell production (53). Moreover, exosomes originating from cells derived from the cardiosphere are transported toward macrophages, allowing cardioprotection in cases of acute myocardial infarction (54, 55). Unraveling the multifaceted contributions of exosomes to safeguarding endothelial integrity could lead to the development of innovative interventions aimed at a spectrum of endothelium-associated disorders.

## 3 Oxidative stress and endothelium damage

### 3.1 Oxidative stress is a multicompartmental phenomenon originating from different sources

**Figure 3:**
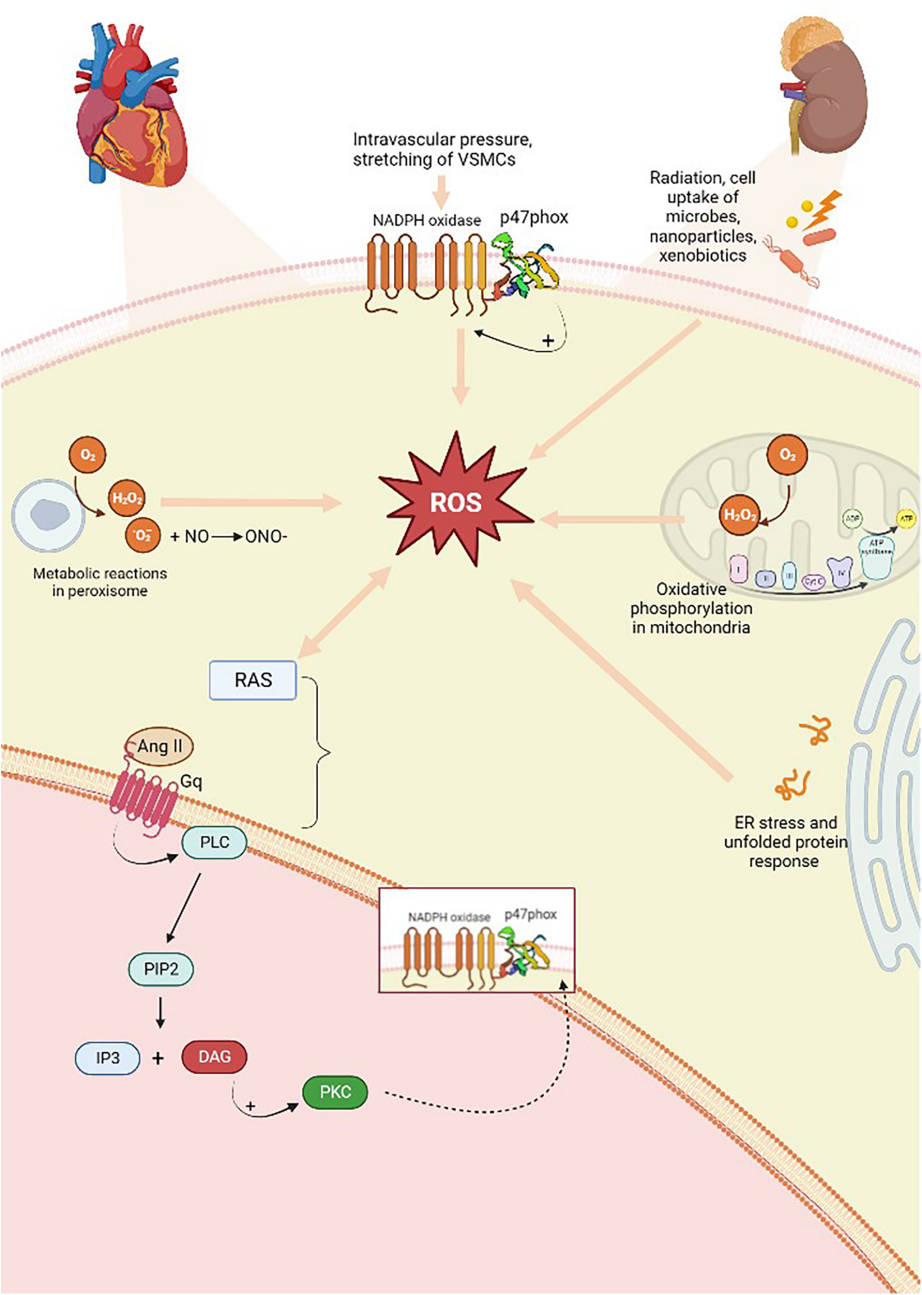
Overproduction of free radicals is responsible and originates from several sources. There is a two-way relationship between ROS production and RAS activation; the Ang II produced activates AT1 (Gq) receptors, which, downstream of the pathway, through the action of DAG, predicts the activation of p47phox, a subunit of NOX, which is also capable of activating the enzyme. ROS, reactive oxygen species; RAS, renin–angiotensin system; AT1, Ang II type 1 receptors; PLC, phospholipase C; PIP2, phosphatidylinositol-4,5-diphosphate; DAG, diacylglycerol; IP3, inositol 1,4,5-trisphosphate; PKC, protein kinase C. Created with BioRender.com.

Oxidative stress occurs when there is large-scale production of ROS and/or an underperforming antioxidant system. These processes contribute to the onset of hypertension in animal models. Experimental evidence has shown that the overproduction of oxygen and nitrogen free radicals (RNS) is responsible for endothelial dysregulation. ROS are produced from molecular oxygen; as free radicals, they are characterized by at least one unpaired electron, which causes their high reactivity. Examples of ROS include O_2_^−^, hydrogen hyperoxide (H_2_O_2_), hydroxyl, peroxyl, and alkoxyl radicals, singlet oxygen, peroxynitrite, ozone, and hypochlorous acid (56). Subjects with hypertension show significantly higher levels of O_2_^−^ and H_2_O_2_ than healthy people. The former is characterized by a short half-life and contributes to vasodilation inactivation. The latter possesses a longer half-life, diffuses more readily, and, in contrast to O_2_^−^, acts as a vasodilator (57). The resulting reaction originates from the interaction of a free radical with a non-radical species; therefore, a cascade of reactions is created that leads to the formation of other reactive compounds. As an example, this kind of reaction occurs in the lipid peroxidation mechanism, where reactive radicals attack the lateral chains of fatty acids (58). ROS are primarily produced in the cardiovascular and renal systems, whereas at the cellular level, they are formed by the mitochondria in the respiratory chain and by phagocytic cells through the enzyme nicotinamide adenine dinucleotide phosphate oxidase (NADPH oxidase, NOX), of which there are several isoforms. VSMCs, endothelial cells, and fibroblasts express NOX-1, NOX-2, NOX-4, and NOX-5 (59). The first two are involved in hypertension onset, as the produced radicals promote inflammation, endothelial dysfunction, lipid peroxidation, increased contractility, and vascular remodeling (56, 59, 60). NOX is stimulated when, under conditions of increased intravascular pressure, there is stretching of VSMCs and endothelial cells, resulting in ROS production. Oxidative stress is implicated in the increase of renin–angiotensin system (RAS) signaling, and the activity of RAS also enhances ROS production. In fact, Ang II is a promoter of oxidative stress; it binds to AT1 receptors and, via G-proteins, activates signaling, in which the secondary messenger, diacylglycerol, contributes to the activation of the phagocytic NOX p47phox, also known as neutrophil cytosol factor 1 (NCF1), a 47 kDa cytosolic subunit (Figure 3) (61). NCF1 with neutrophil cytosol factor 2 (NCF2) and a membrane-bound cytochrome b558 are decisive for NOX activation. In addition, the NCF1 gene interacts with other NOX subunits with significant roles in innate immunity, produces ROS, reduces the gravity and duration of infections and autoimmune diseases, and is involved in T-cell activation. On the cellular level, oxidative damage subsequently occurs, leading to endothelial dysfunction and, thus, hypertension (62). In addition, other factors contribute to ROS-dependent hypertension, such as salt, growth factors, ET-1, immune factors, and aldosterone. Binding to mineralocorticoid receptors, specifically, increases ROS production in endothelial cells and in VSMCs through non-genomic and genomic pathways, activating both NOX1 and NOX4. In fact, signaling of the AT1 receptor is involved in aldosterone-induced oxidative stress, and mineralcorticoid receptor inhibition attenuates ROS generation dependent on Ang II, proving the link between both systems. Endothelial ET-1, NOX1, and NOX2 overexpression in mice is associated with oxidative stress and hypertension. Ang II and ET-1, via G-protein-coupled receptors, activate growth factor receptors including IGF-1, PDGF, and EGF, thereby increasing redox-dependent vascular processes such as vascular remodeling, inflammation, medial hypertrophy, and fibrosis (63). Other cellular sources of endogenous ROS include the endoplasmic reticulum (ER) and peroxisomes (64, 65). However, air pollutants, radiation, and xenobiotics also represent essential sources of ROS (66).

### 3.2 The antioxidants in the endothelial barrier protection

Antioxidants are substances that prevent, decrease, and repair damage caused by ROS. They are able to inhibit the production of radicals in cells and facilitate their removal, thereby repairing the oxidative damage. The antioxidants can originate from endogenous sources, such as superoxide dismutase (SOD), glutathione peroxidase (GPx), and catalase, or they can be derived from foods (56). The establishment of elevated pressure, in addition to the aforementioned causes, is caused by an imbalance between the free radical production and the insufficient ability to counteract their damage using antioxidants (67). Thus, the lack of balance between the antioxidant enzyme activity and the pro-oxidant system results in an excessive increase in oxidative stress that, in turn, results in the manifestation of a dysfunctional endothelium. In this regard, Brunelli et al. (68) demonstrated that at a level of 150 mmHg, reactive oxygen metabolite values remained constant while antioxidant capacity decreased. The reduced antioxidant levels can lead to vascular endothelial cell damage, and in conditions such as preeclampsia during pregnancy, for example, the defensive ability of the total plasma antioxidant barrier is unable to overcome oxidative stress. In a meta-analysis conducted by Taravati and Tohidi (69), it was observed that in pregnant women with preeclampsia, the level of the lipid peroxidation product, malondialdehyde (MDA), was heightened compared with that with a normal pregnancy. The isoforms of SOD prevent excessive generation of ROS at the level of respiratory chain complexes I and III in the mitochondria and also protect against the pathological reduction in NO levels (70). Vascular endothelial cells possess a smaller number of mitochondria, but endothelial dysfunction is still associated with oxidative stress originating from the mitochondria (71). Indeed, the mitochondria influence vascular function through various mechanisms, such as heightened ROS production in the bloodstream and aldehyde dehydrogenase (ALDH) deficiency. In particular, ALDH-2 is a crucial factor for the physiological maintenance of endothelial cells and mitochondria due to its double role in the detoxification of xenobiotics or reactive aliphatic aldehydes and the bioactivation of organic nitrates. Lipid peroxidation and consequent toxic aldehydes are harmful to the inner mitochondrial membrane, as they result in mtDNA damage, apoptosis, mitochondrial permeability, and an impaired electron transport chain (ETC). Therefore, ALDH deficiency is correlated with increased mitochondrial ROS and vascular dysfunction (72).

### 3.3 Relationship between oxidative stress and inflammation, both causes of arterial hypertension

**Figure 4:**
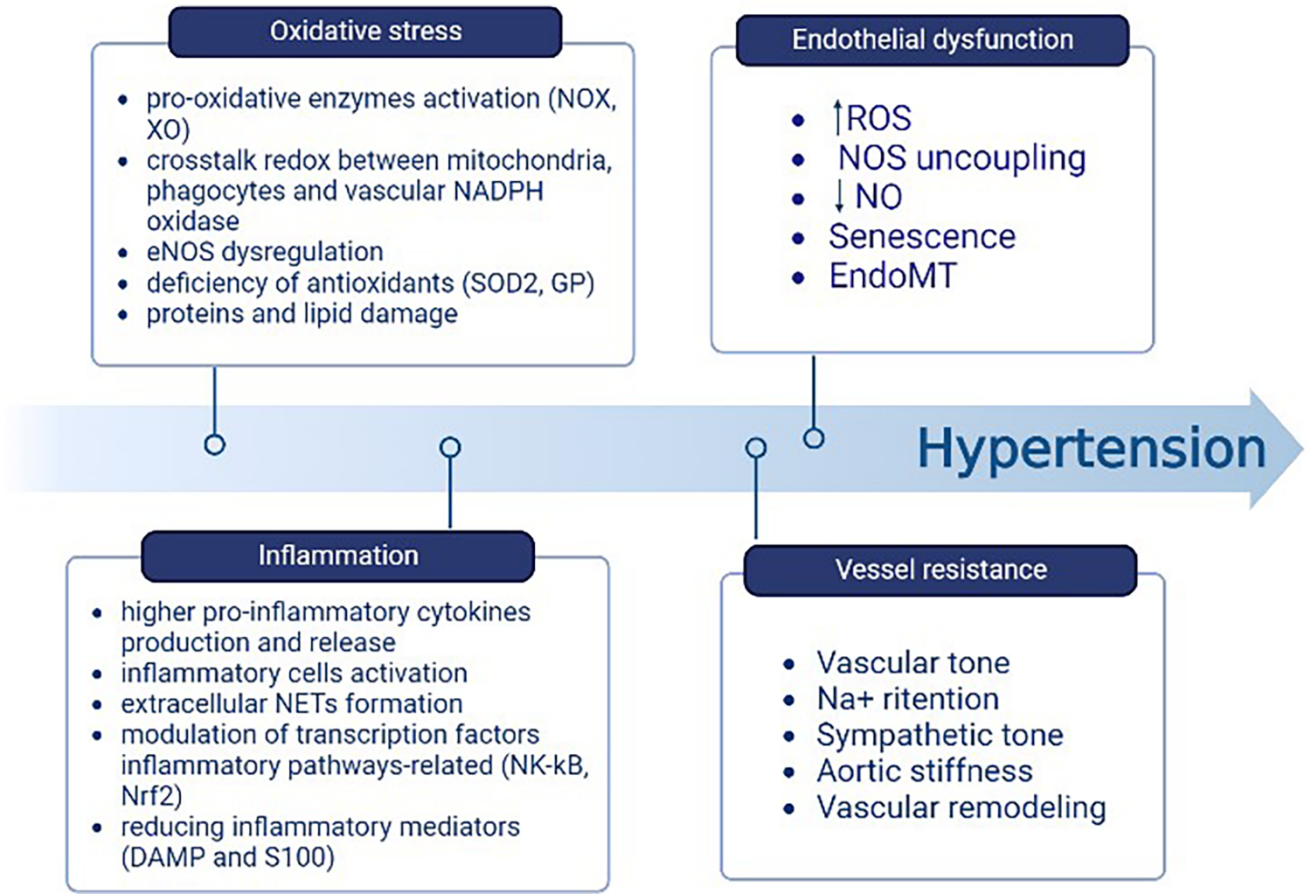
Components contributing to the elevation of BP include oxidative stress and inflammation. One follows the other through the relationship between mtROS, the activation of NLRP3 and subsequent accumulation of damaged mitochondria, the H_2_O_2_ passage from the mitochondrion to the cytoplasm, and the inflammatory cells activation. Created with BioRender.com.

The mitochondria produce an important rate of ROS in the ETC during oxidative phosphorylation and through oxidant enzymes associated with ROS systems in other cellular organelles that act as signaling agents in processes such as inflammation or cell death (56). There is a relationship between the increase in pro-inflammatory cytokines produced by blood cells and the amount of mitochondrial-derived ROS (mtROS), as well as the NLRP3 inflammasome activation and the overproduction of cytokines themselves. This leads to the subsequent accumulation of compromised mitochondria, which drives to the production of ROS in greater quantities. Inflammasomes cause greater permeability to pass H_2_O_2_ through the mitochondrial membrane as it becomes more permeable. Once it reaches the cytoplasmic compartment, H_2_O_2_ contributes to activating the inflammatory cells and triggering the pro-inflammatory pathways (56). ET-1, through the stimulation of its receptor, mediates inflammation and oxidative stress. The inhibition of this receptor has proven to be an efficacious method for the elimination of inflammatory cells (56). In addition, ROS influence inflammatory processes through the formation of extracellular neutrophil traps (NETs); they also regulate the modulation of transcription factors involved in inflammatory pathways, such as nuclear factor erythroid factor 2-related 2 (Nrf2) and nuclear factor-kappa B (NF-kB). Furthermore, ROS decrease the levels of inflammatory mediators such as DAMP and S100 (73). Thus, the oxidative stress and inflammation pathways synergize and interpenetrate with each other (Figure 4) (74).

Different studies showed that microbiota alteration can influence inflammation and substance production, which, if not balanced, could cause endothelium damage and increase arterial stiffness and blood pressure (75). Intermittent fasting, alternating eating periods with fasting periods, influences the microbiota’s composition and activity, indirectly impacting hypertension. This cyclical diet leads to the remodeling of the intestinal microbiota, thereby promoting the growth of beneficial bacteria that produce metabolites such as short-chain fatty acids (SCFA), decreasing systemic inflammation, and enhancing metabolic health (76). Furthermore, the caloric restriction resulting from the intermittent fasting practice leads to weight loss, adipose tissue reduction, better regulation of adipokine secretion, and therefore better insulin sensitivity (77). This interconnection suggests that diet may affect cardiovascular health through changes in gut microbiota and metabolite production; future research is essential to assume the underlying mechanisms.

## 4 From eNOS uncoupling to hypertension: several molecular mechanisms contribute to the pathology establishment

The altered NO production may be involved in the essential hypertension pathogenesis, as human studies showed that an increase in the mean diurnal BP corresponded with a reduced urinary excretion of nitrate, an index of endogenous NO generation. In fact, in patients with untreated essential hypertension, nitrate excretion was lower than that in healthy subjects (15). Among the mechanisms responsible for NO deficiency in hypertension is its destruction by O_2_^−^. By reacting with the NO molecule, O_2_^−^ produces peroxynitrite, which interacts with the amino acid tyrosine and, through a nitration reaction, forms nitrotyrosine, an indicator of nitration levels in patients (78). Several enzyme systems produce ROS. Specifically, O_2_^−^ is produced by NOS, uncoupled eNOS, xanthine oxidase, cyclo-oxygenase (COX), and the enzyme of the mitochondrial respiratory chain (79). Under pathological conditions, NOX is the main cause of the ROS increase, thus leading to a decrease in BH4; consequently, the electrons are not transported to the N-terminal oxygenase domain of either eNOS monomer. As a result, BH4 no longer performs its role as a cofactor, promoting the normal dimerization of eNOS (80). Among ROS, peroxynitrite is mainly responsible for the oxidation of both BH4 and BH2. The latter competes with the former for interaction with eNOS. It does not act as a cofactor itself but causes the enzyme to uncouple (81). The BH4 oxidation by peroxynitrite induces the eNOS uncoupling, which leads to the O_2_^−^ production rather than NO. Various potential factors could lead to eNOS uncoupling: arginine deficiency, protein–protein interaction other than phosphorylation, S-glutathionylation, or acetylation of eNOS. Cigarette smoking is one factor that triggers the acytelation reaction. This causes oxidative stress, accompanied by a decrease in silent information regulatory protein 1 (SIRT1), acetylation of eNOS, and a reduction in NO levels (82). The altered L-arginine/NO pathway also contributes to NO deficiency in hypertension, and a wealth of data attests to the association between L-arginine and the essential hypertension onset (24). In fact, L-arginine transport appears altered in both normotensive and hypertensive patients with a predisposition to essential hypertension for a genetic reason. Furthermore, the offspring of patients with essential hypertension have a reduced capacity to respond to ACH stimuli due to a defect in the NO pathway. The supplementation of L-arginine improves endothelial dysfunction in hypertension (26). In the study conducted by Kimura et al., (83), hypertensive rats characterized by a decreased renal mass demonstrated a reduced NO-mediated relaxation, thus confirming the link to an abnormality at the level of the arterial endothelium. In a study conducted on Dahl hypertensive rats treated with high amounts of salt, in the mesenteric arterioles, the expression of eNOS mRNA was downregulated (84). One of the most well-studied experimental models of hypertension induction in rats, which was first developed by Selye (85) in 1943, involved the administration of deoxycorticosterone acetate (DOCA) and 1% NaCl for 7 weeks. This model has been shown to cause eNOS dysfunction and elevate blood pressure by up to 187–130 mmHg, as compared to the 110–80 mmHg observed in control rats. A study conducted on DOCA salt hypertensive mice has shown decreased levels of the limiting enzyme of the sepiapterin conversion to BH4, sepiapterin reductase (SPR), in aortic endothelial cells compared with the endothelium-denuded aortic ones. This demonstrated an SPR-specific deficiency for endothelium (86). In addition, in hypertensive rats treated with DOCA, the phosphorylation of eNOS in the mesenteric arterioles was downregulated, leading to a decrease in the NO/cGMP pathway at the level of the mesenteric arteries (87).

## 5 Hypertension treatment: old friends and new players

### 5.1 Overview of antihypertensive therapies

**Figure 5:**
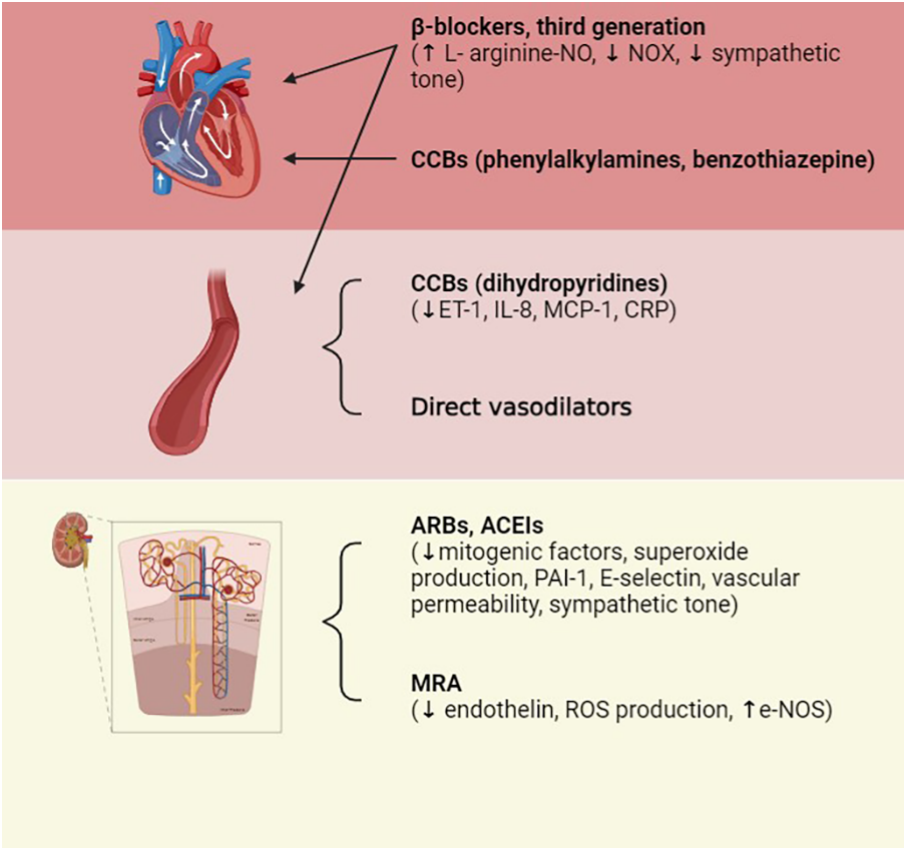
Pharmacological treatments of hypertension. Therapeutic treatment involves actions on multiple targets: on the RAS system by acting on the Ang-converting enzyme in order to reduce its level or by blocking AT1 receptors; on vessels via direct vasodilation; and on atrioventricular conduction or diuresis, thus reducing electrolyte levels. ACEIs, ACE inhibitors; ARBs, Ang II receptor blockers; CCBs, calcium channel blockers; ET-1, endothelin-1; IL-8, interleukin-8; MCP-1, monocyte chemoattractant protein-1; CRP, C-reactive protein; PAI-1, plasminogen activator inhibitor-1. Created with BioRender.com.

In clinical practice, the role of antihypertensive drugs that serve to improve endothelial dysfunction is crucial and enacted through multiple mechanisms, such as counteracting aortic stiffness, oxidative stress, inflammation, EndoMT, and altered vascular tone (88). The pharmacological treatment of arterial hypertension involves possible actions on different pivotal points that, in their own ways, result in the elevation of blood pressure; the mechanisms exploited involve interventions on the RAS system [angiotensin-converting enzyme inhibitors (ACEIs), angiotensin II receptor blockers (ARBs)], vasodilation induced by direct vasodilators [calcium channel blockers (CCBs) such as dihydropyridine], the reduction in atrioventricular conduction (beta-blockers, CCBs such as phenylalkylamine, benzothiazepine), or diuretic actions capable of reducing electrolyte levels, particularly Na^+^ (including loop diuretics, thiazides, and potassium sparing). To induce additive pressure lowering, the current monotherapy is usually combined with a second agent that can block compensatory responses to the initial one, and this drug is represented by the low-dose thiazide diuretic (89). The drug categories that result in the lowering of BP due to the improvement of endothelial function include angiotensin II receptor blockers and angiotensin-converting enzyme inhibitors, confirming that Ang II is responsible for endothelial dysfunction. In fact, the binding of AT1 receptors increases SOD and plasminogen activator inhibitor (PAI-1) production, inflammation, vascular permeability, and sympathetic tone. Ang II also causes aldosterone secretion, sodium and water retention, myocyte hypertrophy, and fibrosis (90). The tight relationship between endothelial dysfunction and hypertension represents a precursor of small vessel disease at the vital organ levels, which include the heart, kidneys, and brain. This supports the hypothesis that endothelial dysfunction has an important impact on the process of systemic vascular remodeling initiated by hypertension and, in general, by other cardiovascular risk factors (91). Mineralocorticoid receptor antagonists (MRAs) are recommended for the management of resistant hypertension. The mineralocorticoid receptor (MR) stimulates ROS formation in endothelial cells and reduces NO production and availability; furthermore, aldosterone potentiates the signaling processes of Ang II in VSMCs. The association of MR block and ACE inhibition in heart failure allows for a decrease in ROS production and the improvement of both endothelial function and left ventricle remodeling (92). Among the drug categories of interest, CCBs have been shown to have pleiotropic effects able to improve endothelial function and reduce central aortic pressure (93). However, the effects of these drugs are not directly related to their well-known mechanism of action but rather to a secondary mechanism that involves the reduction of ET-1, C-reactive protein (CRP), and the production of monocyte chemoattractant protein-1 (MCP-1). Third-generation beta-blockers, such as nebivolol, characterized by dual actions on β and α receptors, also enhance endothelial function through NO-dependent vasodilatation and antioxidant effects. In addition, ET-1 receptor antagonists are attracting growing interest for the prevention of vascular remodeling, endothelial dysfunction, and possible organ damage that may occur in hypertensive disease (94). The use of combinations of multiple fixed-dose antihypertensive drugs also improves patient compliance by eliminating issues related to forgetfulness or difficulty taking multiple tablets each day, thus allowing the therapeutic target to be finally reached. Drug combinations with similar action mechanisms or clinical effects should be avoided, as should the use of two drugs that target the same BP control system; in fact, these will be less effective and harmful if used concomitantly, as may be the case for the combination of ACE inhibitors and sartans, which can increase the risks of cardiovascular and renal problems. However, a combination of K-sparing diuretics such as amiloride and thiazide results in an equivalent CCB treatment and causes fewer side effects than thiazide alone (glucose intolerance and hypokalemia). The concomitant use of different classes of diuretics (such as thiazide, K-sparing diuretics, and/or loop diuretics) and CCBs (such as dihydropyridine and non-dihydropyridine) may be combined. Other associations with benefit are ACEIs and CCBs and ARBs or ACEIs and diuretics. The use of ACEIs and CCBs, or ARBs and diuretics, has proved more efficient than the beta-blocker with diuretics, a therapy that could result in diabetes in susceptible individuals (7, 8). Figure 4 represents an overview of antihypertensive therapies (Figure 5).

### 5.2 Nutraceuticals: natural antioxidants and valid aids for hypertension treatment

In recent years, nutraceuticals and functional foods have increasingly been incorporated into adjunctive therapies for treating various cardiovascular disorders. Specifically, nutraceuticals consist of concentrated forms of bioactive substances obtained by foods, which are used at dosages above those obtainable from them (95). Based on current guidelines, depending on the lipid profile and the associated cardiovascular risk of the patient, a correct strategy must be adopted for that specific group of patients (96, 97). In those already treated, a combined therapy is used, and the non-pharmacological treatment is always associated with lifestyle modification. This is recommended for all types of patients, while lipid-lowering agents are recommended only under certain conditions, as taken from the 2019 ESC/EAS guidelines on dyslipidemia treatment (98). Nutraceuticals and functional foods have beneficial effects that help in achieving therapeutic targets regarding total cholesterol concentration. Natural products, in fact, improve not only the lipid profile but also insulin resistance, glucose concentration, inflammation, oxidative stress, vascular stiffness, and blood pressure. They are safe products, assuming that the production quality is guaranteed as well as the absence of additives and impurities, such as the presence of citrinin in red rice. Due to the growing attention regarding the properties of nutraceuticals, we can talk about nutrivigilance, thanks to the efforts of experts and manufacturers who have improved the monitoring, safety, and reporting process of any side effects (96). Nutraceuticals can prevent the development of hypertension in prehypertensive patients by avoiding the need for the prescription of blood-pressure-lowering drugs, and they can be used as a supplement for hypertensive patients close to their optimal goal. The use of nutraceuticals has been shown to result in a lower hypertensive status and a decreased risk of cardiovascular disease and metabolic syndrome (89). In fact, several trials have shown that certain flavonoids are useful for the natural treatment of mild hypertension. Among the widely studied and well-known molecules playing this role are coenzyme Q10, melatonin, L-arginine, vitamin C, and omega-3 PUFAs (99), as well as flavonoids from beans, tea, red wine, cocoa (*Theobroma cacao* L. bean seeds possess more phenolic compounds and higher antioxidant activity) (100), garlic (although it is known to enhance the effects of oral antidiabetic drugs and anticoagulants), extra virgin olive oil, *Orthosiphon stamineus* Benth., and monacolins of red yeast extract. Carrizzo et al. (101) demonstrated that *Morus alba* L. extract causes NO-mediated endothelial vasorelaxation through the increased phosphorylation of eNOS. The beneficial activity of *Morus alba* L. seems to occur through the activation of protein kinase RNA-like ER kinase (PERK) and HSP90, important stress sentinel proteins, and chaperones. In addition, the studies conducted by Badran et al. on ethanolic extracts of *Origanum majorana* L. and by Anwar and colleagues on *Salvia fruticosa* Mill. have highlighted the ability to cause vasodilation and relaxation of the thoracic aortic vessels in rats. This occurs through the endothelium-dependent process that activates the PI3-K/eNOS/cGMP phosphoinositide 3-kinase pathway (102, 103). However, this type of treatment needs to be accompanied by a balanced and healthy lifestyle, including exercise, a proper diet, with the most widely recommended option being the Mediterranean diet, and the loss of excess weight. These different contributions effectively reduce cardiometabolic risk factors and the Framingham Risk Score (104). However, although the selective action of antioxidants in counteracting the production of ROS is an interesting therapeutic strategy for the treatment of hypertension, in patients with various cardiovascular risk factors and with probable, however realistic, irreversible oxidative damage, the only antioxidant therapy may not be sufficient to reverse the damage (9).

### 5.3 Nutraceuticals actives

**Figure 6:**
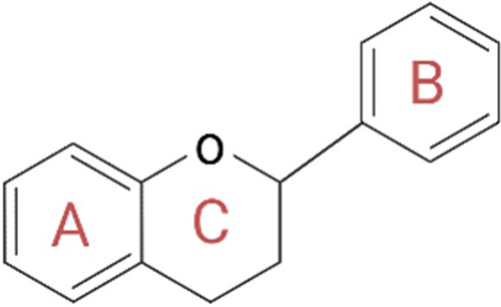
General structure of flavonoids. The structure is critical for the biological functions performed by flavonoids, such as their cytotoxicity and antioxidant and antiproliferative activities. In fact, according to the different accessory functional groups, these polyphenols are divided into subfamilies (flavanones, flavones, flavonols, isoflavones, flavanols, and anthocyanidins), depending on the degrees of unsaturation and oxidation of the oxygenated heterocycle. Created with BioRender.com.

Polyphenols play a key role in the therapeutic action based on nutraceutical use. These constitute a broad and heterogeneous class of phytochemicals that include flavonoids, stilbenes, phenolic acids, and lignans (105). Polyphenols are produced by herbs as secondary metabolites to protect themselves from the damage caused by oxidative stress, ultraviolet light, and extreme temperatures. As active ingredients in nutraceuticals, polyphenols are primarily involved in the prevention of cardiovascular diseases, which mainly include blood pressure, endothelial function, platelet function, and circulating lipids (106). Their ability to protect against ROS is mainly related to their structure consisting of one or more phenolic groups, which explains the growing and justified interest in the polyphenol role in the prevention of chronic diseases related to oxidative stress (107). Among the aforementioned polyphenols, flavonoids are probably the most representative group. They are low-molecular-weight polyphenolic molecules, mainly consisting of two benzene rings (A and B) bonded together by three carbons and an oxygen atom to form a pyran (C) (Figure 6). Based on the different functional groups acting as accessories to the main scaffold, flavonoids are divided into subfamilies such as flavanones, flavanols, flavonols, flavones, isoflavones, and anthocyanidins, depending on the degrees of oxidation and unsaturation of the oxygenated heterocycle. The flavonoid structure has a key role in their biological functions, such as their cytotoxicity as well as their antioxidant and antiproliferative activities. Again, depending on their structure, they are also able to expose zinc metalloproteinase, as in the case of the ACE enzyme (108).

### 5.4 A new nutraceutical squad: *Citrus bergamia* (Risso et Poiteau), *Orthosiphon stamineus* Benth., *Hibiscus sabdariffa* L., *Berberis aristata* L., and *Olea europaea* L.

The anti-inflammatory and antioxidant properties of main polyphenols from plants such as *Citrus bergamia* (Risso et Poiteau), *Orthosiphon stamineus* Benth., *Hibiscus sabdariffa* L., *Berberis aristata* L., and *Olea europaea* L. have been studied. The phytocomplex resulting from this combination, called BergaPress [developed at H&D s.r.l. Bianco (RC)], was analyzed for its polyphenol content: *Hibiscus sabdariffa* L. contained more than 5%, *Orthosiphon stamineus* Benth. contained sinensetin at a concentration greater than 0.20%, *Berberis aristata* L. contained berberine at 87.76%, *Olea europaea* L. contained oleuropein at 20%, and the bergamot polyphenolic fraction contained neohesperidin (>11%), naringin (>11%), neoeriocitrin (>9%), bruteridin (>2%), and melitidine (>1%) (109). The multiple beneficial properties exerted by a single plant/herb, through the reduction of oxidative stress, the modulation of different molecular pathways, and the regulation of various energy and cellular mechanisms are listed in Table 1.

**Table 1:** Different mechanisms of action exerted by the plant/herb listed below to counteract oxidative stress, inflammatory status, and metabolic and endothelial dysfunctions.

Plant/herb	Active principle/specialty	Mechanism of action	Side effects
*Citrus bergamia* (Risso et Poiteau) (109)	BPF, BEO-NVF naringin, melitidine, bruteridin, neoeriocitrin, rutin, neohesperidin, poncirin.	– Enhancement of NO and PGI2 production, with a consequent vasodilating effect. – Inhibition of tyrosine nitration. – Reduction in lipid peroxidation biomarkers (TBARS). – Improved SOD, GPx, and GSTP1 activity. – Reduced expression levels of LOX-1 and MDA. – Increased potassium regulation due to maintenance of the Na + /K + pump. – Reduced cholesterol levels in serum and tissue via the inhibition of HMG-CoA reductase.	Lack of significant side effects.
		– Reduction in total cholesterol, LDL, IDL, and TG (PAP inhibition) levels in plasma. – Reduction in fasting plasma glucose levels. – Increased HDL levels. – Reduction in inflammation and liver fibrosis through PARP inhibition. – Increased PKB phosphorylation.	
*Orthosiphon stamineus* Benth. (110–112)	Rosmarinic acid (RA), sinensetin (SIN), eupathrin (EUP), 30-hydroxy-5,6,7,40- tetramethoxyflavone (TMF).	– NO-mediated vasorelaxation. – VOCC and ROCC channel inhibition. – Opening of KCa, KV, KATP, Kir channels. – Muscarinic and beta-adrenergic receptor ago nist. – ACE inhibitor. – Alpha-adrenergic receptor antagonist. – Probable involvement of PGI2.	There are no known side effects, contraindications, or interactions with other drugs or medications.
*Berberis aristata* L. (113–119)	Berberine (BBR).	– ACE inhibitor. – Activation of via ROS/Erk1/2/iNOS. – NO-mediated vasorelaxation and promotion of vasodilation. – M endothelial receptor stimulation. – Reduced expression of iNOS. – Reduced oxidative stress. – LOX-1 production, reduced expression of ox- LDL, and increased hepatic LDLr. – Reduction in intracellular Ca + in VSMCs – Inhibition of PP2A pathway. – Maintenance of arterial elasticity. – TRPV4 channel inhibition, VSMC relaxation. – MyD88-TLR4 pathway inhibition and endo- thelial apoptosis. – CXCR4/JAK-2 pathway inhibition, endothelial cell protection. – AMPK pathway activation, RE stress inhibition in endothelial cells. – Decreased NF-kB and TNF-α expression.	No serious adverse effects were found during the treatment of type 2 diabetes, hyperlipidemia, and hypertension. In these studies, BBR treatments had a relatively low cost compared with other drugs and first-line treatments
*Hibiscus sabdariffa* L. (98, 120–124)	Hibiscina, gossypicianin.	– ACE inhibition. – NO-dependent vasodilation. – K + channels activation. – Ca2 + channels inhibition. – Ca2+-calmodulin complex inhibition – Antioxidant and anti-inflammatory effects.	Does not present particular problems and usually does not generate side effects.
*Olea europaea* L. (125–127)	Oleuropein, oleacin, tyrosol, hydroxytyrosol, Tensiofytol ®.	– Antioxidants via stimulation of the NrF2 pathway. – Antiarterosclerotic activity via antiplatelet and antiperoxidative antiplatelet activity. – eNOS upregulation. – Reduction in plasma levels of total cholesterol, LDL, TG, IL-8. – Reduction in LDL oxidation. – Hepatic fat oxidation. – Increased HDL levels. – Anti-inflammatory activity with downregula tion of NF-kB via the inhibited translocation of p65 and p50 (PMA). – Increased LPS-induced NO production via iNOS stimulation. – NO-mediated vasodilation. – Improved endothelial function measured as RHI. – Clostridia XIV abundance inversely correlated with systolic BP values.	No significant side effects but only slight ones including nausea, cramps, diarrhea, or headache. It was observed no mutagenicity evidence *in vitro*, either genotoxicity evidence *in vivo*.

### 5.5 *Citrus bergamia* (Risso et Poiteau)

Bergamot (*Citrus bergamia* Risso et Poiteau) is an endemic species that grows exclusively on the Ionian coast of Calabria. This fruit is distinguished from other fruits of the genus Citrus by its important composition of glycosides and flavonoids (naringin, neohesperidin, neoeriocitrin, rutin, and poncirin) and particularly high juice content (128). Bergamot reduces hunger sensations and has a strong effect on insulin sensitivity, lipid metabolism, and glucose tolerance. The positive influence of bergamot on cardiovascular health is well documented, mostly due to its anti-inflammatory properties that protect the vascular endothelium from ROS (129). The bergamot essential oil non-volatile fraction (BEO-NVF) has been shown to possess significant antioxidant properties; thus, it is considered promising to prevent endothelial dysfunction, proliferation of smooth muscle cells, and inflammation. The antioxidant effect was tested in a neointima hyperplasia experimental model. Mollace et al. (130) demonstrated that the pretreatment of rats with BEO-NVF reduced neointima formation, ROS generation, oxidized-LDL receptor-1 (LOX-1) expression, and the degree of stenosis caused by prior balloon injury in carotid arteries. Bergamot juice can be concentrated using a patented method based on size-exclusion chromatography, filtration on polystyrene gel, and drying of the eluate to obtain a powder enriched in polyphenols, the bergamot polyphenol fraction (BPF) (109). The use of BPF is of great interest because of its demonstrated antihypertensive, anti-dyslipidemic, and antioxidant activities. Mollace R. et al. (131) performed a study on cyclists in order to evaluate how much BPF Gold [BPF at 48.9% in polyphenols: naringin (>14%), neohesperidin (>13%), neoeriocitrin (>13%), bruteridin (>5%), and melitidine (>2%) developed at H&D s.r.l. Bianco (RC)] supplementation was required to improve serum asymmetric dimethyl-arginine (ADMA) concentrations and NO, EndoPAT vascular indices related to endothelium-dependent vasodilation, and muscle oxidative capacity in athletes. Indeed, it is known that performing high-intensity exercise results in an excessive production of free radicals and increased oxidative stress compared with normal conditions. BPF Golf supplementation for 4 weeks was shown to exert a beneficial effect on endothelium-dependent vasodilation at rest, as serum NO levels were found to be increased compared with the levels prior to supplementation. The underlying mechanisms potentially responsible for the BPF Gold vasoprotective effect include NO production and hepatic fat oxidation in the skeletal muscle. The mechanism underlying the reduction in inflammation and liver fibrosis is based on the inhibition of poly(ADP-ribose) polymerase-1 (PARP1) by BPF. The additional vasoprotective effect of BPF is closely related to its antioxidant properties. It has been revealed to decrease lipid peroxidation biomarkers (TBARS) and MDA, enhance the activity of GPx, SOD, and glutathione S-transferase P1 (GSTP1) enzymes, and inhibit nitrotyrosine levels (132). BPF is able to decrease LOX-1 expression levels, which are highly modulated in the establishment and progression of endothelial dysfunction toward atherosclerosis. The BPF vasoprotective action is also related to the increased phosphorylation of PKB, which provides protection against atherogenic vascular damage (109). Stanzione et al. (133), by means of an interesting study on SHR stroke-prone animals, demonstrated the beneficial action of BPFs. Primary brain endothelial cells were placed in a culture environment and exposed to high saline concentrations (NaCl 20 nM) for 72 h in the presence or absence of BPF. The results obtained showed that BPF is able to inhibit the molecular mechanisms underlying salt-induced endothelial cell damage, confirming that it could be a valuable adjuvant in the treatment of vascular disorders. In fact, in agreement with others, this study found that the supplement of BPF decreases mitochondrial oxidative stress, rescuing mitochondrial function itself, cell viability, and angiogenesis, in contrast to the effects of a high salt concentration.

### 5.6 *Orthosiphon stamineus* Benth.

*Orthosiphon stamineus* (OS) Benth., also known as Misai Kucing, is a medicinal herb in the *Lamiaceae* family, native to Southeast Asia. It is traditionally known for its use in the treatment of hypertension. Studies have demonstrated that its leaves possess diuretic, antidiabetic, and anticancer effects; renal, gastric, and hepatic protective activities; and antioxidant and anti-inflammatory activities (134). OS leaves contain high levels of phenolic acids, such as rosmarinic acid (RA), and flavonoids, such as eupathrin (EUP), sinensetin (SIN), and 30-hydroxy-5,6,7,40-tetramethoxyflavone (TMF). Due to these properties, OS has been identified as a potential candidate for drugs with ACE inhibitory activity. Indeed, Shafaei et al. (108) evaluated ACE inhibitory activity *in vitro* using different OS leaf extracts. The study showed that the mechanism of action of flavonoid compounds depends on their ability to bind the zinc ion at the active site of the ACE enzyme, thus inhibiting it. Through docking studies, it was possible to explore the quality and quantity of the bonds that the single molecules were able to create in the enzymatic pocket. It emerged that EUP is able to contribute to the inhibition of the enzyme as it can form seven hydrogen bonds with the active site of the enzyme itself. Other studies have shown that the vasorelaxant activity of the herb is due to SIN. In this regard, Yam et al. (110) used the precontracted aortic ring assay *in vitro*, demonstrating that the mechanism underlying the SIN vasorelaxant effect involves the NO/sGC/cGMP pathways, potassium and calcium channels, and β-adrenergic and muscarinic receptors.

#### 5.6.1 Evidence of different mechanisms of action of *Orthosiphon stamineus* Benth.

The antihypertensive activity of OS is due to its vasodilator and diuretic activities, the latter probably due to its binding with the renal adenosine A1 receptor (135). Yam et al. (136) studied the mechanism of action that characterized the therapeutic effect of OS using the chloroform fraction of methanolic extract at 50% OS on the aorta of Sprague Dawley rats. OS was shown to possess multiple mechanisms. The vasodilation effect occurs through the antagonization of aortic ring contraction in both an indirect and endothelium-independent direct manner. In this latter case, the vasodilation effect was far greater than that in the former. The vasorelaxant action of OS extract was reduced via L-NAME, indicating that the NO/cGMP pathway is implicated in the vasorelaxant mechanism of this herb. To evaluate the possible involvement of PGI2, the action of OS was compared with that of indomethacin, a non-selective COX inhibitor. However, the low degree of inhibition demonstrated by the extract suggested that this type of pathway was not primarily involved in the vasodilation process. The underlying mechanism was found to involve other actions on potassium and calcium channels and β-adrenergic and muscarinic receptors. Upon interaction with the receptor, phenylephrine, an adrenergic agonist, produces diacylglycerol, activates PKC, and enables Ca^2+^ influx through ROCC, the receptor-activated calcium channel. Calcium influx through ROCC and VOCC, voltage-operated calcium channels (137), will cause ion release from the sarcoplasmic reticulum through the activation of IP3 and ryanodine receptors. OS extract leads to a reduction in intracellular calcium levels and subsequent relaxation via ion influx inhibition through VOCC, as the nifedipine action, and reduces vasoconstriction caused by phenylephrine, demonstrating its involvement in IP3 and/or ryanodine receptors. Furthermore, OS induces relaxation, modulating the opening of four potassium channels present in arterial smooth muscle cells: voltage-dependent K^+^ channels (KV), Ca^2+^-activated K^+^ channels (KCa), rectifying K^+^ channels (Kir), and ATP-sensitive K^+^ channels (KATP) (138). In agreement with these results, Manshor et al. (111) showed how OS induces vasorelaxant effects through the release of NO from the endothelium by inhibiting the release of intracellular Ca^2+^ and/or through the blockade of ROCC channels and the involvement of α1-receptors. In addition, in this study, it was found that PGI2 release might not participate in vasodilation; however, in the group treated with methanolic extract, the improvement might have been due to the fact that PGI2 is released continuously, as evidenced by its effects on platelet cAMP. In addition, OS evidently consists of compounds that behave as agonists for muscarinic and beta- adrenergic receptors, since the extract-induced vascular relaxation was affected by the respective antagonists, atropine and propranolol, with which pretreatment was carried out (136). *Orthosiphon stamineus* Benth., combined with policosanol, berberine, red yeast rice extract, coenzyme Q10, and folic acid, demonstrates potential for controlling blood pressure in those with normal BP, high-normal BP, and grade I hypertension. Through 24-hour ambulatory BP monitoring (ABPM), average reductions of more than 15 mmHg in systolic BP and 10 mmHg in diastolic BP were demonstrated following the administration of the nutraceutical (139).

### 5.7 *Berberis aristata* L.

Cultivated and known in the West as Indian Berberis or barberry, *Berberis aristata* L. is a thorny shrub of the *Berberidaceae* family. This herb is native to the mountainous areas of India, Nepal, and Sri Lanka. Its roots, used in Ayurvedic and popular Indian medicine, have curative and purifying virtues. The antibacterial, anti-inflammatory, and antioxidant properties of *Berberis aristata* L. are due to berberine (BBR), an important isoquinoline alkaloid found in many herbs in the genera *Berberis* and in the Chinese herb *Coptis chinensin* Franch (140). BBR, used extensively in traditional Chinese medicine, has demonstrated the ability to inhibit the growth of various bacteria, viruses, fungi, protozoa, chlamydia, and helminths. It is also beneficial for cancer, cerebrovascular disease, atherosclerosis, obesity, and respiratory disorders; protects vascular endothelial function; reduces the progression of hypertension; and restores insulin secretion by inhibiting oxidative stress. In SHR rats, BBR was shown to be able to inhibit cholinesterase and decrease BP by directly bonding the M receptor of vascular endothelial cells without dilating the blood vessels (113). It also improves endothelial function by inhibiting ER stress and the expression of NF-kB, Toll-like receptor 4 (TLR4), TNF-α, and myeloid differentiation primary response 88 (Myd88). Tian H. et al. (141) studied how BBR infusion into the paraventricular nucleus (PVN), a relevant site for the central regulation of blood pressure, could inhibit hypertension in two-kidney, one-clamp (2K1C) rats. In the study, BBR was found to decrease sympathetic activity and hypertension via the ROS/Erk1/2/iNOS pathway. On the PVN level, radicals activate the sympathetic system during hypertension via the p44/42 MAPK pathway. ERK1 (p44 MAPK) and ERK2 (p42 MAPK) are activated due to the increased presence of ROS (140). Chronic infusion of berberine into the PVN reduces the expression levels of iNOS, highlighting the close relationship between pressure elevation and increased iNOS expression. BBR has a hypotensive effect through the inhibition of cGMP production and the release of NO and ACE in vascular tissues. It inhibits oxidative stress and decreases the expression of oxidized LDL (oxLDL) and oxidized-LDL lectin-like receptor 1 (140). BBR has an interesting stabilizing effect on the LDL cholesterol receptor (LDLr) on the hepatic cell surface, the same mechanism of action as that of PCSK9 inhibitors. The combination of berberine and monakolina has led to the improvement of BP via flow-mediated dilation in humans (89). BBR improves vascular stiffness and aging via the blockade of transient receptor potential vanilloid 4 (TRPV4) channels and the reduction in intracellular Ca^2+^ levels in VSMCs (142).

### 5.8 *Hibiscus sabdariffa* L.

*Hibiscus sabdariffa* L. is an herbaceous plant that can be annual or perennial, or a woody-based subshrub, which belongs to the *Malvaceae* family and is native to Malaysia and India (143). The phytocomplex that characterizes *H. sabdariffa* L. includes polysaccharides, organic acids, and flavonoids, including anthocyanins such as delphinidin-3-sambubioside, called hibiscina, and cyanidin-3-sambubioside, also known as gossypicianin, which are responsible for the plant vasorelaxant activity (120). The different chemical structures of the polyphenols influence their bioavailability and activity, determining the general hypotensive effect characteristic of *H. sabdariffa* L. For example, ponidin, cyanidin, catechin, and epicatechin, by means of catechol-O-methyltransferase, generate compounds analogous to apocynin, a vasoactive compound. Furthermore, quercetin derivates, such as 4-methylcatechol (4MC), dihydroxyphenylacetic acid (DHPA), and 3-(3-hydroxyphenyl) propionic acid (3HPPA), demonstrate hypotensive effects when they act simultaneously. The vasodilator action takes place through the metabolites of bioactive compounds at the colonic level, of which 3HPPA is the most effective compound (144). *H. sabdariffa* L. extract is traditionally used as an antihypertensive agent, as evidenced by several *in vivo* studies on 2K1C and clinical rats. It has been demonstrated that the antihypertensive action of *H. sabdariffa* L. extracts used in volunteers is due to the inhibition of the ACE enzyme by the anthocyanins hibiscina and gossypicianin (121). *H. sabdariffa* L. also has an endothelium-dependent vasodilator effect involving the PI3-kinase/Akt pathway and subsequent NO release (122) and an endothelium-independent effect via the inhibition of Ca^2+^-channels and the Ca^2+^–calmodulin complex and activation of potassium channels. Haji Faraji and Haji Tarkhani (123) assessed the effect of *H. sabdariffa* L. tea on 54 patients with moderate hypertension and, within 12 days, revealed a reduction in BP, with an elevation in BP observed 3 days after stopping the treatment. Other studies conducted by Ali et al. (145) assessed the antihypertensive effects of *H. sabdariffa* L. and the anthocyanin isolated from it in CKD in Wistar rats by preventing systolic BP elevation following adenine treatment, while Joven et al. (146) evaluated the efficacy of the polyphenolic compound in both SHR rats and patients with metabolic syndrome. Serban et al. (147) analyzed the aqueous extract effect of the HS dried calyx on SHR rats. In addition to reducing systemic blood pressure, it decreased left ventricular mass in a dose-dependent manner and increased the surface area and myocardial capillary length density. Elkafrawy N. et al. (148) studied the synergistic effects of *O. europaea* L. and *H. sabdariffa* L. in Egyptian patients with first-degree hypertension. It was found that this composition, at both high and low doses, was able to reduce mean arterial pressure in a manner comparable with the ACE inhibitor captopril.

### 5.9 *Olea europaea* L.

*Olea europaea* L. is a typical fruit tree found in the Mediterranean region and is probably native to Asia Minor. Olive leaves, particularly in Mediterranean countries, are commonly used as a traditional remedy to counteract several diseases. The geographical origin of olive drupes, the cultivar, the ripening stage, the irrigation, and oil extraction conditions influence the concentration of the extract. *O. europaea* L. leaf extract is rich in monounsaturated fatty acids (MUFAs), particularly oleic acid; 2% of the total weight of the oil unsaponifiable fraction is composed of minor components, which are heterogeneous compounds chemically not related to fatty acids (149). The nutraceutical properties of *O. europaea* L. are correlated with secoiridoid oleuropein (OL) and derivatives, p-hydroxyphenyl ethanol (also known as tyrosol), and 3,4-dihydroxyphenyl ethanol (or hydroxytyrosol, HT) (150). Olive leaf extract (OLE) has antihypertensive and antioxidant effects. OL and HT have antioxidant effects, working as metal chelators and scavengers of free radicals. Due to their catechol structure, they are able to scavenge peroxyl radicals and disrupt peroxidative chain reactions, producing species with a stable structure (151). They inhibit the oxidation of HDL and LDL *in vitro* and *in vivo* through the repression of radical reactions. However, this beneficial effect cannot definitively be attributed to the antioxidant action against ROS due to excessively low plasma concentrations (approximately 1–10 uM), as compared with the analyzed concentrations of the blood antioxidants vitamin C, E, and glutathione (152). The potential mechanism underlying the effect of HT could be the autoxidation of the hydroxyl groups of phenolic compounds in the intracellular compartment. Subsequent to the increased production of ROS, cells stimulate the expression of antioxidant enzymes through the activation of the transcription of NrF2. This appears to be stimulated by olive oil, HT, tyrosol, and OL (153). The combined effect of 20 mg of HT and 100 mg of oleuropein per day led to a decrease in BP and the protection of the cardiovascular system by the enhancement of endothelial function and the synthesis of NO (153). Either in cellular or animal models, OL and HT have been found to increase the production of NO by upregulating eNOS. This effect has proved useful in the resolution of the pathological components of metabolic syndrome.

#### 5.9.1 *Olea europaea* L. extract improves endothelial function acting on multiple compartments

OLE reestablishes the phosphorylation of aortic eNOS at the levels of Thr-495 and Ser-1177, increasing the activity of eNOS itself. It reduced the mRNA levels of NOX-1 and NOX-2, resulting in downstream reductions in NADPH oxidase activity and aortic superoxide levels (22). Studies performed on SHR have demonstrated improvements in vascular function following treatment with OLE, which is able to decrease BP in essential hypertension patients and, albeit slightly, blood glucose and calcemia (154). Susalit et al. (125), though a double-blind, controlled, parallel, randomized trial using olive leaf extract vs. captopril, evaluated the extract tolerability and the antihypertensive effect in patients with hypertension at the first stage. They found that 500 mg of the extract administered twice daily reduced BP to a degree comparable with the ACE inhibitor at a dose of 12.5–25 mg twice daily. Colica et al. (155) demonstrated that the administration of 14 mg/day of HT for 3 weeks among 28 healthy volunteers conferred a strong antioxidant effect on the plasma through the increased expression of thiol proteins and SOD and a decreased concentration of MDA. The activity of the active ingredients, oleuropein and oleacin, is probably associated with the ACE inhibitory action. In particular, oleuropein was responsible for the vasodilatory effects of endothelium-independent and reduced sinus node function and the contractile response of the atria in Guinea pigs (156). In fact, the study conducted by Romero et al. (22) demonstrated that OLE directly and reversibly inhibited the L-type calcium channel. In addition, a significant increase in red fluorescence was found in the aorta of the SHR group incubated with dihydroethidium (DHE), indicating increased vascular O_2_^−^ levels. The *in vivo* administration of OLE treatment effectively counteracted the observed increase in oxidative stress, as evidenced by the decrease in serum levels of MDA. This effect was observed in both hypertensive and diabetic rats treated with oleuropein and in rats fed a high-carbohydrate and high-fat diet and treated with an olive leaf extract enriched in oleuropein. In addition, a consequence of a protracted hypertensive state establishment is the development of hypertrophy on the cardiac and renal levels, as was observed in SHRs that presented increased indices of left ventricular and renal weights compared with the normotensive WKY rat group. In contrast, chronic treatment with OLE significantly reduced this impairment. In addition, the antioxidant effects, the reduction in heart rate, and protection from NO reduction provided by OLE appear to play a preventive role in the morphological changes in SHR rats treated with OLE. Treatment with the extract of *O. europaea* L. demonstrated, in a mouse model of isoproterenol-induced myocardial infarction, that it prevented the inflammation state and decreased infarct size, enhancing the shortening and ejection fraction. *O. europaea* L. plays its protective roles through the involvement of signaling pathways of Akt/eNOS and ERK1/2-Prdx1 and 2 (157). OLE protective effects on vascular inflammation could be associated with TLR4 expression due to the p38 MAPK activation and decreased ROS production; moreover, OLE modulates TLR4 signaling, reducing the phosphorylation of IκBα and pro-inflammatory cytokine production. In SHR rats, the phosphorylation of ERK1/2 and TLR4 levels in the aorta was found to be higher than that in WKYs. Treatment with OLE reduces the levels of phosphorylation and TLR4. In addition, OL and HT inhibit LPS-induced NF-kB activation in endothelial cells. LPS stimulates TLR4 expression in vessels, which contributes to increased BP, inflammation, and NOX-dependent O_2_ production (158). The production of ROS and the activation of the MAPK signaling pathway lead to an increased TLR4 expression and subsequent mRNA stabilization in human aortic smooth muscle cells. Cirmi et al. (159) focused on oleacin (OLC), an abundant secoiridoid present in the *O. europaea* L. phytocomplex. The data revealed the intense molecular anti-inflammatory and antioxidant OLC activities, which determine ROS level reduction in the intracellular compartment and a decrease in COX-2, NO, and PGE2 levels augmented via exposure to LPS in THP-1-derived macrophages. In fact, OLC inhibits the CD14/TLR4/CD14/MyD88 axis and activates NF-κB, thus modulating inflammatory signaling pathways. OLE is able to reduce the IL-6 and IL-8 levels in human coronary artery endothelial cells (HCAECs) via endogenous serum amyloid A (SAA) inflammation, probably as a result of NF-kB p65 phosphorylation. OLE treatment also resulted in the reduced expression of the pro-adhesive molecules E-selectin and VCAM-1 in HCAECs and human umbilical vein endothelial cells (HUVECs) following pro-inflammatory stimulation with LPS, TNF-, and PMA (160).

## 6 Discussion

In this review, we highlighted the importance of endothelial function as the central hub of vascular health. Endothelium integrity has a crucial role in the preservation of balance and the release of vasoactive molecules that, depending on various conditions, result in vasodilation or vasoconstriction. Conversely, an altered endothelium leads to the loss of this balance; on the molecular level, a series of events occur that rely on the chief molecule of endothelium-dependent relaxation, NO, including the uncoupling of NOS and oxidative stress. The prolongation of such events unquestionably leads to endothelial damage and the debilitation of vascular tone, eventually resulting in increased peripheral resistance. These changes represent the critical point that establishes arterial hypertension, which is identified in the pressure increase on the blood vessels, resulting in increased afterload by forcing the heart to exert more effort in order to compensate for the resistance created by the metarterioles. There are several ways to improve a hypertensive state: pharmacologically, one can act on the RAAS system, on the blood vessels via vasodilation, or on the heart via a reduction in atrioventricular conduction or through a diuretic action that can lower electrolyte levels and, above all, Na ^+ ^. In addition to drug therapy, however, the use of nutraceuticals with antioxidant actions, including bergamot polyphenolic fraction, *Orthosiphon stamineus* Benth., *Hibiscus sabdariffa* L., *Berberis aristata* L., and *Olea europaea* L., is of clear scientific interest. These nutraceuticals have some mechanisms of action in common, including the inhibition of ACE, the enhancement of NO-mediated vasodilation, interactions with ion channels including the blocking of calcium channels and opening of potassium channels with consequent membrane hyperpolarization, anti-inflammatory effects with reductions in synthesis and release of pro-inflammatory cytokines, and enhancement of endogenous antioxidant actions exerted by different enzymes, such as SOD, GPx, and GSTP. The phytocomplexes of these plants were titrated into polyphenols and used in the formation of a new product, BergaPress, in order to exploit the actions characterizing these different components to obtain a synergy that could be employed in the prevention of BP or as a supplementary adjuvant to BP therapy.

## 7 Evidence gaps and future perspectives

The present review has extensively examined, through highlighting the many preclinical and clinical studies, the mechanisms underlying endothelial dysfunction and hypertension and the current conventional and innovative therapeutic strategies to counteract them.

Nevertheless, evidence gaps exist; indeed, although there are several epidemiological and natural history studies that suggest that the early treatment and prevention of hypertension could improve outcomes, further clinical studies are needed to provide more comprehensive and wide-ranging guidance. In particular, further evidence regarding the hypertensive target organ damage and cardiovascular disease risk and outcomes is required, which should include the investigation of the effects of more extensive combined therapy, broadening awareness of hypertension as a risk factor, and the benefits of treatment. The therapeutic approach to optimization involves continuous, focused research that examines the prevention of hypertension development and the benefits of maintaining a lifetime low BP. Among these, the analysis of genetic expression, epigenetic modulation, and proteomics could add significant knowledge about the identification of new treatment targets through understanding the underlying pathophysiological mechanisms. Given the current evidence regarding the role of antioxidants of natural origin in the prevention of endothelial dysfunction, further clinical trials should be directed toward the development of combined therapies that directly counteract the mechanisms accounting for the development of hypertension and disease progression. Further research using conventional therapy in combination with nutraceuticals could aim to develop clinical and population-based strategies to prevent obesity, improve fitness, and control excessive food intake. There is a need for large-scale clinical studies to evaluate the effects of this strategy, which could have an important impact on public health by significantly reducing cardiovascular risk.
